# Exploring the effects of skeletal architecture and muscle properties on bipedal standing in the common chimpanzee (*Pan troglodytes*) from the perspective of biomechanics

**DOI:** 10.3389/fbioe.2023.1140262

**Published:** 2023-05-05

**Authors:** Xiao-Wei Xv, Wen-Bin Chen, Cai-Hua Xiong, Bo Huang, Long-Fei Cheng, Bai-Yang Sun

**Affiliations:** State Key Lab of Digital Manufacturing Equipment and Technology, School of Mechanical Science and Engineering, Institute of Medical Equipment Science and Engineering, Huazhong University of Science and Technology, Wuhan, Hubei, China

**Keywords:** common chimpanzee, bipedal standing, musculoskeletal model, Hill-type MTU, constrained optimization, simulation experiments

## Abstract

**Introduction:** It is well known that the common chimpanzee, as both the closest living relative to humans and a facultative bipedal, has the capability of bipedal standing but cannot do so fully upright. Accordingly, they have been of exceeding significance in elucidating the evolution of human bipedalism. There are many reasons why the common chimpanzee can only stand with its hips–knees bent, such as the distally oriented long ischial tubercle and the almost absent lumbar lordosis. However, it is unknown how the relative positions of their shoulder–hip–knee–ankle joints are coordinated. Similarly, the distribution of the biomechanical characteristics of the lower-limb muscles and the factors that affect the erectness of standing as well as the muscle fatigue of the lower limbs remain a mystery. The answers are bound to light up the evolutional mechanism of hominin bipedality, but these conundrums have not been shed much light upon, because few studies have comprehensively explored the effects of skeletal architecture and muscle properties on bipedal standing in common chimpanzees.

**Methods:** Thus, we first built a musculoskeletal model comprising the head-arms-trunk (HAT), thighs, shanks, and feet segments of the common chimpanzee, and then, the mechanical relationships of the Hill-type muscle-tendon units (MTUs) in bipedal standing were deduced. Thereafter, the equilibrium constraints were established, and a constrained optimization problem was formulated where the optimization objective was defined. Finally, thousands of simulations of bipedal standing experiments were performed to determine the optimal posture and its corresponding MTU parameters including muscle lengths, muscle activation, and muscle forces. Moreover, to quantify the relationship between each pair of the parameters from all the experimental simulation outcomes, the Pearson correlation analysis was employed.

**Results:** Our results demonstrate that in the pursuit of the optimal bipedal standing posture, the common chimpanzee cannot simultaneously achieve maximum erectness and minimum muscle fatigue of the lower limbs. For uni-articular MTUs, the relationship between muscle activation, relative muscle lengths, together with relative muscle forces, and the corresponding joint angle is generally negatively correlated for extensors and positively correlated for flexors. For bi-articular MTUs, the relationship between muscle activation, coupled with relative muscle forces, and the corresponding joint angles does not show the same pattern as in the uni-articular MTUs.

**Discussion:** The results of this study bridge the gap between skeletal architecture, along with muscle properties, and biomechanical performance of the common chimpanzee during bipedal standing, which enhances existing biomechanical theories and advances the comprehension of bipedal evolution in humans.

## 1 Introduction

Apart from modern humans (*Homo sapiens*), there are various other species of primates, such as the common chimpanzee (*Pan troglodytes*), that have acquired the ability of bipedal standing; however, they cannot stand fully upright (Jenkins, 1972). As our closest living relative (Goodman, 1999; Waterson et al., 2005), common chimpanzees (chimps) share with us the post-cranial features related to orthograde modes of locomotion (Hunt, 1991) while lacking the human-like skeletal architecture that aligns the shoulder, hip, knee, and ankle joints in the sagittal plane (Thorpe et al., 1999; Payne et al., 2006b; Pontzer, 2018). Circumscribed to the distally oriented long ischial tubercle and the almost absent lumbar lordosis (Robinson, 1972; McHenry, 1975), chimps are unable to extend their hip joint so that they stand bipedally in a bent-hip, bent-knee manner, a position in which the center of mass (CoM) is located anterior to the hip (Sockol et al., 2007). When a human stands upright, the CoM, hip, knee, and ankle joints approximately line up (Neumann, 2013), diminishing the necessity for the lower-limb muscles to be activated (Pontzer et al., 2009). In the case of a chimp, muscle activation of the hind limbs is required to generate muscle forces and joint moments on account of balance when it comes to standing on two feet. However, how do chimps coordinate the relative positions of their shoulder, hip, knee, and ankle joints? How is the muscle activation of the hind limbs assigned in pursuit after maintaining balance? How do biomechanical factors such as skeletal architecture and muscle properties affect the erectness of standing? Although the bipedal locomotion pattern of chimps has been studied since 1944 (Elftman, 1944), few studies have explored this topic in depth.

The road to uncovering the aforementioned questions is paved with challenging puzzles. Although laboratory-based experiments have been widely conducted in the bipedal walking or running of chimps (Demes et al., 2015; Thompson et al., 2015; Kozma et al., 2018), their application remains unfeasible in bipedal standing since it is barely possible to keep untrained chimps standing bipedally for a sufficient amount of time (Doran, 1992a; Doran, 1992b; Hunt, 1992; Hunt, 1994). Training may not be a feasible alternative either because trained primates have been proven to develop changes in the musculoskeletal structure (Nakatsukasa, 2004). Modeling and simulation can not only overcome these problems but can also provide internal parameters that are difficult to measure. However, existing musculoskeletal models (Yamazaki, 1985; O'Neill et al., 2013; O'Neill et al., 2015; O'Neill et al., 2018; O'Neill et al., 2022; Sellers et al., 2013) are still far from effective for investigating the biomechanical performance of chimps during bipedal standing. Yamazaki (1985) measured the net joint moments of chimps during bipedal locomotion under controlled conditions and derived the corresponding muscle forces using computer simulations. However, Yamazaki assumed constant values of the muscle moment arms that apparently vary with joint angles and did not specify whether anatomical or physiological cross-sectional areas were used to estimate muscle forces from stresses. O'Neill et al. (2013, 2015, 2018, 2022) quantified the variation in moment arms and muscle forces of hind limb muscles with joint angles during bipedal locomotion in chimps through modeling and simulations on the OpenSim platform, combined with detailed muscle–tendon parameters. However, their model merely contained the pelvis and hind limbs and could not predict the maximum erectness that chimps can achieve during bipedal standing and the corresponding posture. Sellers et al. (2013) established a whole-body model of the common chimpanzee on the GaitSym platform to explore changes in performance, such as footfall sequences, locomotion velocity, and energy expenditure during quadrupedal locomotion within the domain. However, their model simplified the pattern of muscle activation according to the motor function, which is solely applicable to rhythmic movements.

Numerical optimization is recognized as a viable technique for predicting animal behavior (Pandy, 2001), where it is crucial to translate appropriate biomechanical metrics into optimization objectives (Lee and Umberger, 2016). Experimental studies have indicated that the larger the hip and knee joint angles, that is, the greater the erectness, the smaller the activation volume of the hind limb muscles during bipedal walking in chimps (Pontzer et al., 2014). This implies that erectness and muscle activation can be considered as the objective function for optimization. Furthermore, muscle fatigue, as a pivotal biomechanical indicator, can be regarded as an objective function that characterizes muscle activation (Crowninshield and Brand, 1981). The constrained optimization is thus applicable for exploring the bipedal standing postures (BSPs) of chimps and corresponding biomechanical factors. This methodology thoroughly assesses multiple biomechanical factors of the skeletal muscles, such as the isometric force, deformation, and muscle fatigue. To the best of our knowledge, this approach is the first-ever attempt to predict the optimal bipedal standing posture of chimps and is a valuable complement to existing biomechanical theories.

This study aimed to investigate the biomechanical effects of skeletal architecture and muscle properties on bipedal standing in chimps. First, a musculoskeletal model based on anatomical data comprising the head–arms–trunk (HAT), thighs, shanks, and feet segments of the common chimpanzee was developed. Second, the static relationships among the Hill-type muscle–tendon units (MTUs) in bipedal standing were deduced. Next, the equilibrium constraints and the optimization objective were set up, which transformed the investigation into a constrained optimization problem. Subsequently, thousands of simulations of bipedal standing experiments in chimps were conducted. Finally, the optimal posture that simultaneously maximizes erectness and minimizes muscle fatigue of the hind limbs was determined *via* numerical searching within the domain, and MTU parameters including muscle activation, muscle length, and muscle force were drawn. In addition, the biomechanical effects under investigation were stipulated by the Pearson correlation analysis of the outcomes from simulating experiments.

## 2 Materials and methods

### 2.1 Musculoskeletal modeling

In the strictest sense, the analysis of bipedal standing in chimps should be conducted in three dimensions. However, reckoning with the reality that the mechanical behaviors of bipedal standing in chimps mainly occur in the sagittal plane, we subsequently generated the musculoskeletal model in the sagittal plane.

#### 2.1.1 Segmental and skeletal properties

The body of the common chimpanzee can be represented as seven segments: the HAT (including the pelvis), bilateral thighs (including femurs), bilateral shanks (including tibias and fibulas), and bilateral feet.

Without loss of generality and to ensure computational efficiency, the following hypotheses were presented: 1) the thigh length and femur length were the same; 2) the shank length was the same as the tibia length; 3) the foot was subdivided into the forefoot, midfoot, and rearfoot, and the midfoot and rearfoot were integrated as one rigid piece; 4) the hip joint connecting the HAT and the thigh, the knee joint connecting the thigh and the shank, the ankle joint connecting the shank and the foot, and the metatarsophalangeal (MP) joint connecting the forefoot and midfoot were all simplified as smooth hinges; 5) the ground reaction force was evaluated as a resultant force and its acting point at the foot was the center of pressure (CoP); and 6) the external force only involved the gravitational force and the support force from the ground, and friction was neglected.

**FIGURE 1:**
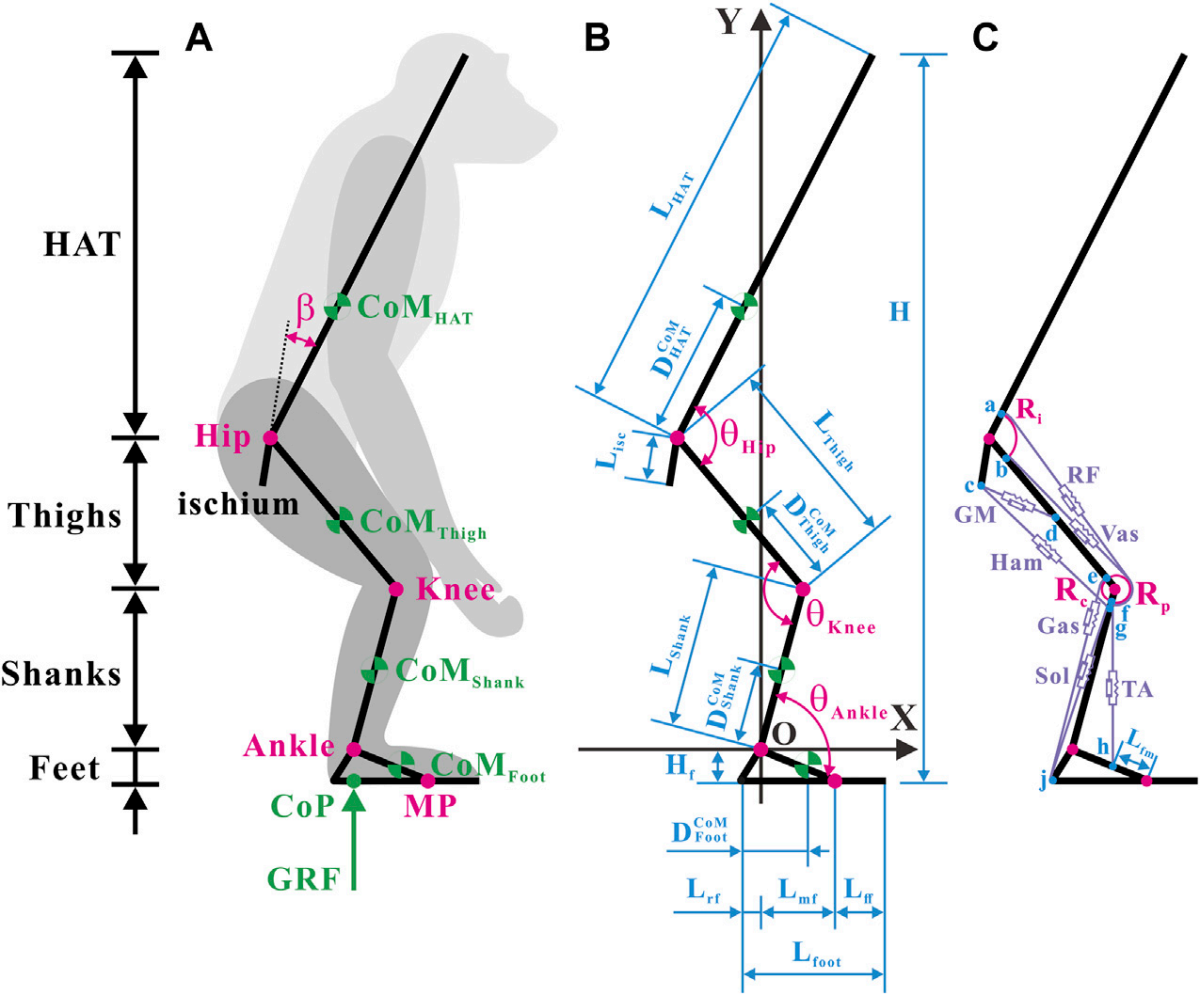
Musculoskeletal model of a bipedally standing common chimpanzee in the sagittal plane. **(A)** The skeletal elements include seven segments (HAT, bilateral thighs, bilateral shanks, and bilateral feet) and the ischium, with mobile articulations at the hip, knee, ankle, and metatarsal-phalangeal joints. **(B)** Annotations of segmental parameters (length of each segment and sub-segment, and position of CoM in each segment) and joint angles. **(C)** The muscular elements include the primary extensors and flexors of each lower limb [gluteus maximus (GM), hamstrings (Ham) (biceps femoris long head, semimembranosus, and semitendinosus), vastus (Vas), rectus femoris (RF), gastrocnemius (Gas) (gastrocnemius lateralis and gastrocnemius medialis), soleus (Sol), and tibialis anterior (TA)]. Blue dots: a) anterior-inferior iliac spine; b) the combined origin point of the vastus intermedius, vastus lateralis, and vastus medialis; c) midpoint of the most superior and to the most inferior points of the ischial tuberosity face; d) midpoint of the posterior femur shaft; e) medial and lateral femoral condyles and knee capsule; f) patella; g) fibular head; h) proximal end of the metatarsal I; j) distal end of the rear foot. Adapted from Diogo et al. (2013), Thorpe et al. (1999), Payne et al. (2006b), Myatt et al. (2011).

The segments, CoMs, joints, segmental angles, and ground reaction force (GRF) are shown in Figure 1A. Annotations of the segmental parameters and joint angles are shown in Figure 1B. The precise values of the segmental parameters are listed in Supplementary Table S1, and their sources are disclosed in Section 2.1.3. The positive direction of the joint angles was defined as the hip joint extension, knee joint extension, and ankle joint extension. Given that the hip joint of chimps cannot be entirely extended owing to the orientation and length of the ischium (Kozma et al., 2018), the acute angle between the ischium and HAT was fixed as a constant β .

Considering computational efficiency, a global Cartesian coordinate system with the rotation center of the ankle joint as the origin was created.

#### 2.1.2 Muscle geometry and Hill-type MTU

Conscientious observations of the anatomy of chimps (Thorpe et al., 1999; Payne et al., 2006b; Myatt et al., 2011; Diogo et al., 2013) have revealed that the extensor and flexor muscles, which primarily produce forces and joint moments in the sagittal plane in each hind limb, incorporate a total of 10 muscles in seven muscle groups, as shown in Figure 1C. It should be noted that the gluteus maximus (GM) only comprises the gluteus maximus ischiofemoralis according to Stern (1972), Tuttle et al. (1978), and Lieberman et al. (2006).

After careful estimation of bone landmarks and muscle maps of chimps (Thorpe et al., 1999; Payne et al., 2006b; Myatt et al., 2011; Diogo et al., 2013), the points of origin and insertion for each muscle were designated, located as close to the center of the attachment area as possible, as shown in Figure 1C. In accordance with the attachment points and wrapping paths of muscles, the functional relationship between the lengths and moment arms of muscles with respect to joint angles can be verified. In particular, because the femoral condyle contour in chimps is significantly more circular than that in humans (Heiple and Lovejoy, 1971), the femoral condyle was approximated as a circular arc in the sagittal plane. The tibia was presumed to move wholly along the femoral condyle contour, and the patella was presumed to move completely along the femoral–tibial condyle contour (O'Neill et al., 2013). Consequently, the moment arms of the rectus femoris (RF) and vastus (Vas) around the knee joint were constant ( $R_{p}$ ), and the moment arm of gastrocnemius lateralis and gastrocnemius medialis (Gas) around the knee joint was constant ( $R_{e}$ ) when tangential to the femoral condyle. With reference to the measurements (Thorpe et al., 1999; Payne et al., 2006a), the moment arm of RF about the hip joint was assumed constant ( $R_{i}$ ).

**FIGURE 2:**
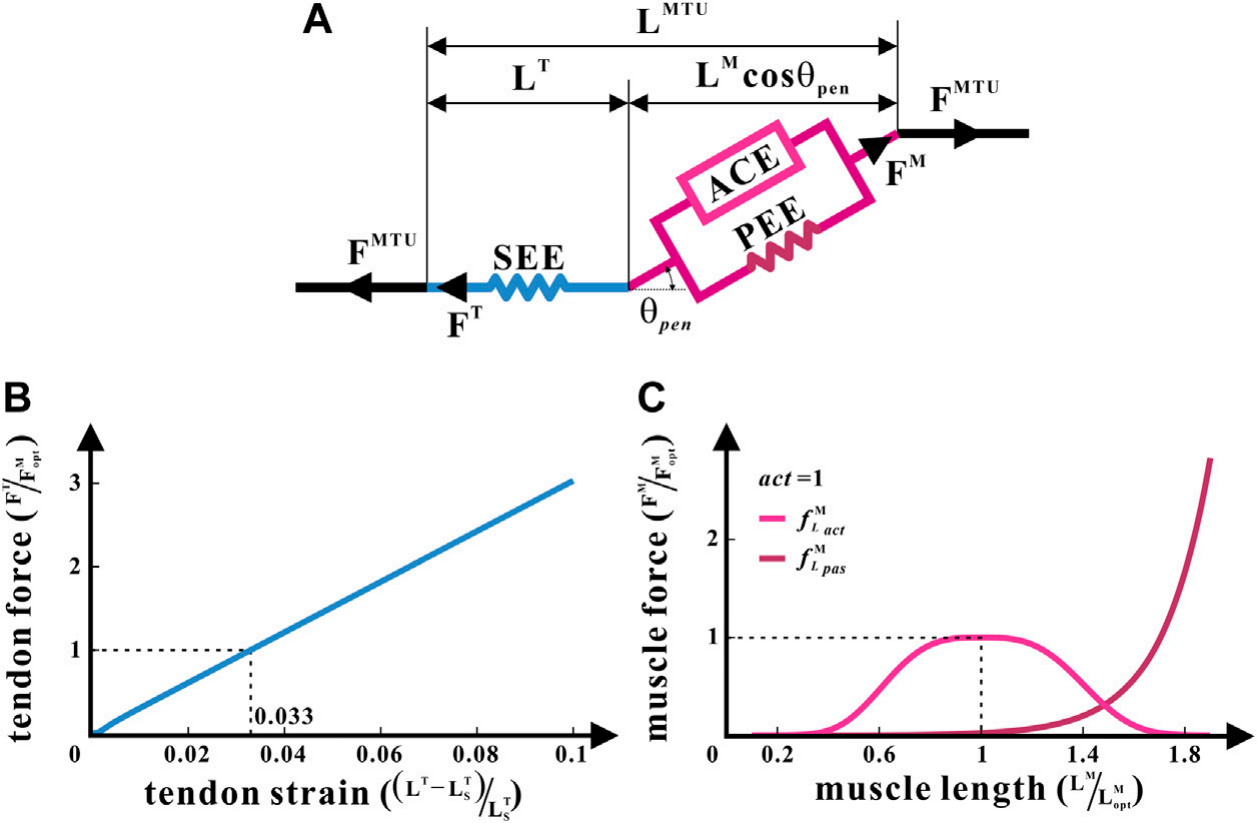
Hill-type MTU of the common chimpanzee. **(A)** Structure of the Hill-type MTU. **(B)** Non-linear relationship between the tendon force and tendon strain for SEE. **(C)** Non-linear relationships between the relative muscle force and relative muscle length, respectively, for ACE and PEE.

A generic Hill-type model (Zajac, 1989) was applied to enumerate metrics such as the lengths and forces of muscles and tendons in each hind limb under isometric contraction as the joint angle changed (Sawicki and Khan, 2015), as illustrated in Figure 2. It was the posture of bipedal standing that this study intended to analyze. Therefore, the muscle contraction velocity $v^{M}$ was approximated to zero, and the bipedal standing of the common chimpanzee was statically analyzed.

The Hill-type model treats each muscle as an MTU. Every MTU consists of two portions: a part associated with the traits of the muscle fibers and another part equated to the tendon. They are in series with each other, between which is the pennation angle $\theta_{pen}$ . The muscle part is composed of an active contractile element (ACE) arranged in parallel with a passive non-linear elastic element (PEE), while the tendon part consists of a series elastic unit (SEE). Because no $\theta_{pen}$ of any MTU in chimps is greater than 30° , which means $\cos\theta_{pen}\approx 1$ (Thorpe et al., 1999), all $\theta_{pen}$ were approximated to zero (Payne et al., 2006). Furthermore, the muscle and tendon in MTUs were confined to the following force and length relationships:

$$\begin{array}{c} F^{MTU}=F^{M}=F^{T} \end{array},$$ (1)

$$\begin{array}{c} L^{MTU}=L^{M}+L^{T} \end{array},$$ (2)

where $F^{MTU}$ denotes the force of MTU, $F^{M}$ denotes the muscle force, $F^{T}$ denotes the tendon force, $L^{MTU}$ denotes the length of MTU, $L^{M}$ denotes the muscle length, and $L^{T}$ denotes the tendon length.

The biomechanical characteristics of each MTU were confirmed by five internal parameters: the mass of MTU ( $m^{MTU}$ ), optimal fiber length ( $L_{opt}^{M}$ ), physiological cross-sectional area ( PCSA ), optimal isometric muscle force ( $F_{opt}^{M}$ ), and tendon slack length ( $L_{S}^{T}$ ), the values of which are shown in Table 1 and explained in Section 2.1.3.

**TABLE 1:** Hill-type MTU parameters.

	$m^{MTU}$ (g)	PCSA (cm^2^)	$L_{opt}^{M}$ (mm)	$F_{opt}^{M}$ (N)	$L_{S}^{T}$ (mm)
Gluteus maximus	300	27.9	101	837	35.4
Biceps femoris (long head)	85	5.1	157	153	123.6
Semimembranosus	67	4.0	158	120	122.4
Semitendinosus	100	3.6	260	108	74.9
Rectus femoris	93	11.3	78	339	260.4
Vastus	455	44.7	95.3	1,341	196.6
Gastrocnemius lateralis	67	7.9	80	237	206.6
Gastrocnemius medialis	90	10.6	80	318	207.2
Soleus	128	22	55	660	212.1
Tibialis anterior	50	5.3	88	159	137.9

With the aforementioned parameters, $F^{M}$ and $F^{T}$ can be addressed by the following formula:

$$\begin{array}{c} F^{M}=F_{opt}^{M}∙\left(act∙f_{L}^{M}{}_{act}+f_{L}^{M}{}_{pas}\right) \end{array},$$ (3)

$$\begin{array}{c} F^{T}=K^{T}∙\left(L^{T}-L_{S}^{T}\right) \end{array},$$ (4)

where act is the muscle activation between $\left[0,1\right]$ , $f_{L}^{M}{}_{act}$ is the active coefficient of the muscle force–length relationship, $f_{L}^{M}{}_{pas}$ is the passive coefficient of the muscle force–length relationship, and $K^{T}$ is the coefficient of the tendon stiffness. In addition, $f_{L}^{M}{}_{act}$ and $f_{L}^{M}{}_{pas}$ are both functions of $L^{M}$ . $K^{T}$ is a function of $F^{T}$ , and $F^{T}=F_{opt}^{M}$ when LT−LSTLST=0.033 (Zajac, 1989). Subsequently, under circumstances wherein every $L^{MTU}$ has been counted from $\theta_{Hip}$ , $\theta_{Knee}$ , and $\theta_{Ankle}$ , together with the knowledge of $L_{opt}^{M}$ , $F_{opt}^{M}$ , and $L_{S}^{T}$ , all the unknown variables in MTUs can be articulated as functions of $L^{M}$ .

#### 2.1.3 Musculoskeletal dataset

Full sets of anatomical data of the common chimpanzee are limited (Zihlman and Cramer, 1978; Thorpe et al., 1999; Wang and Crompton, 2004; Isler et al., 2006; Kozma et al., 2018), among which source sample Chimp 95 (Thorpe et al., 1999) stands out for integrity. To construct the complete dataset of the skeletal and muscular parameters in need, the anatomical data from other sources were scaled to match that of sample Chimp 95 (Thorpe et al., 1999), depending on the geometric similarity between different individuals of the same species.

The segmental masses of sample Pa1 in Table 3 from Isler et al. (2006) were scaled according to the ratio of the mass of Chimp 95 $m_{Chimp95}$ to that of Pa1 $m_{Pa1}$ for obtaining the corresponding head mass $m_{Head}$ , trunk mass $m_{Trunk}$ , upper arm mass $m_{Uarm}$ , forearm mass $m_{Farm}$ , hand mass $m_{Hand}$ , thigh mass $m_{Thigh}$ , shank mass $m_{Shank}$ , and foot mass $m_{Foot}$ of Chimp 95. The HAT mass $m_{HAT}$ of Chimp 95 was calculated as follows:

$$\begin{array}{c} m_{HAT}=m_{Head}+m_{Trunk}+2\times \left(m_{Uarm}+m_{Farm}+m_{Hand}\right) \end{array}.$$ (5)

The segmental lengths of sample Pa1 in Table 3 from Isler et al. (2006) were scaled according to the ratio of masses as ${\left(\frac{m_{Chimp95}}{m_{Pa1}}\right)}^{\frac{1}{3}}$ for attaining the corresponding head length $l_{Head}$ , trunk length $l_{Trunk}$ , and foot length $l_{Foot}$ of Chimp 95. The HAT length $L_{HAT}$ of Chimp 95 was calculated as follows:

$$\begin{array}{c} L_{HAT}=l_{Head}+l_{Trunk} \end{array}.$$ (6)

The relative position of each segmental CoM of sample Pa1 in Table 3 from Isler et al. (2006) was, respectively, multiplied by the segmental lengths of Chimp 95 worked out previously to attain the corresponding segmental CoM positions of Chimp 95, that is, the distance from the HAT CoM to the hip joint $D_{HAT}^{CoM}$ , the distance from the thigh CoM to the knee joint $D_{Thigh}^{CoM}$ , the distance from the shank CoM to the ankle joint $D_{Shank}^{CoM}$ , and the distance from the foot CoM to the heel $D_{Foot}^{CoM}$ .

The average fibular length ${\bar{X}}_{fibula}$ of samples *Pan troglodytes* in Table 1 from Zihlman and Cramer (1978) was scaled according to the ratio of the tibial length of Chimp 95 $L_{tibia}$ to the average tibial length of *Pan troglodytes* ${\bar{X}}_{tibia}$ for obtaining the corresponding fibula length $L_{fibula}$ of Chimp 95.

The foot length parameters of the chimpanzee species in Table 1 in the paper by Wang and Crompton (2004) were scaled by the ratio of the foot length of Chimp 95 $L_{Foot}$ to that of the species chimpanzee FL for attaining the corresponding foot height $H_{f}$ , rearfoot length $L_{rf}$ , midfoot length $L_{mf}$ , forefoot length $L_{ff}$ , and third metatarsal length $L_{fm}$ of Chimp 95.

To obtain the corresponding ischial length $L_{isc}$ of Chimp 95, the ischial lengths of the 20 samples of *Pan troglodytes* in Supplementary Table S1 from the study by Kozma et al. (2018) were averaged.

The value of β was elicited from the maximum hip angle ( 162° ) in the presence of the dimensionless mechanical advantage of samples of *Pan troglodytes* in Table S1 from Kozma et al. (2018):

$$\begin{array}{c} \beta =180{}^{\circ}-162{}^{\circ}=18{}^{\circ} \end{array}.$$ (7)

The values of $m^{MTU}$ , $L_{opt}^{M}$ , and PCSA were obtained from sample Chimp 95 by Thorpe et al. (1999). The value of $F_{opt}^{M}$ , which characterizes the capacity of muscle force production, can be acquired by multiplying the PCSA with the maximum isometric muscle stress, which was set to 0.3 MPa according to previous studies (Wells, 1965; Thorpe et al., 1999). The value of $L_{S}^{T}$ was obtained using the constrained non-linear optimization function (fmincon) in the MATLAB optimization toolbox (MathWorks, Natick, MA, United States) (O'Neill et al., 2013), following the force and length relationships between muscles and tendons in MTUs (Zajac, 1989; Manal and Buchanan, 2004; Sawicki and Khan, 2015).

### 2.2 Search of solutions

It is well-known that chimps, like any primate, are constrained in their erectness when standing bipedally by their skeletal architecture and muscle properties; for example, they must maintain balance and stability, and their muscles and tendons should not exceed the force ranges (O'Neill et al., 2013). These constraints were translated into numerical boundaries merged with the geometric and mechanical relationships of the musculoskeletal system, as described in Section 2.1. Subsequently, the objective function was interpreted in accordance with the principle of simultaneously maximizing erectness and minimizing the muscle fatigue of the hind limbs. Finally, the constrained optimization was carried out through random initial values and random directions within the domain, and this process was repeated to globally search for the bipedal standing postures (BSPs) of chimps. During the procedure, every set of optimization results was equivalent to one simulation experiment of bipedal standing in chimps.

#### 2.2.1 Biomechanical constraints

To guarantee the stability of bipedal standing, the overall CoM of the common chimpanzee was assumed to be maintained directly above the ankle joint:

$$\begin{array}{c} X^{CoM}=0 \end{array},$$ (8)

where $X^{CoM}$ is the horizontal coordinate of the CoM about the origin, which is a function of $\theta_{Hip}$ , $\theta_{Knee}$ , and $\theta_{Ankle}$ .

To ensure the balance of bipedal standing in chimps, the MTU of the hind limbs must be able to produce the torque desired by the hip, knee, and ankle joints:

$$\begin{array}{c} M_{i}^{need}=M_{i}^{prod}\\ \left(i=Hip,Knee,Ankle\right), \end{array}$$ (9)

where $M_{i}^{need}$ is the required moment of the hip, knee, and ankle joints, and $M_{i}^{prod}$ is the moment produced by the hip, knee, and ankle joints. Referring to Figure 1A, $M_{i}^{need}$ can be expressed as a function of $\theta_{Hip}$ , $\theta_{Knee}$ , and $\theta_{Ankle}$ . Referring to Figure 1C, combined with Section 2.1, $M_{i}^{prod}$ can be, respectively, expressed as functions of $\theta_{Hip}$ , $\theta_{Knee}$ , $\theta_{Ankle}$ , and $L^{M}$ .

#### 2.2.2 Constrained optimization

Considering that chimps seek the maximum erectness and minimum muscle fatigue of hind limbs during bipedal standing, the optimization objective was defined as the square of the ratio of erectness to the muscle fatigue of hind limbs:

Object=Erectness2Fatigue2, (10)

where Erectness is the ratio of height to the full length of the body during bipedal standing:

$$\begin{array}{c} Erectness=\frac{H}{H_{f}+L_{Shank}+L_{Thigh}+L_{HAT}} \end{array},$$ (11)

where H is the height during bipedal standing. Thus, Erectness is a function of $\theta_{Hip}$ , $\theta_{Knee}$ , and $\theta_{Ankle}$ .

The muscle fatigue of hind limbs was manifested as follows (Anderson and Pandy, 2001):

$$\begin{array}{c} Fatigue=\underset{i}{\sum }{\left({act}_{i}\right)}^{2}\\ \left(i=GM,bflh,semimem,semiten,Vas,RF,Gasl,Gasm,Sol,TA\right). \end{array}$$ (12)

Consequently, Fatigue is a function of $L^{M}$ .

To sum up, Object is a function of $\theta_{Hip}$ , $\theta_{Knee}$ , $\theta_{Ankle}$ , and $L^{M}$ .

#### 2.2.3 Data analysis

To obtain the numerical solution of Object that is as close to the global optimal solution as possible, the values of $\theta_{Hip}$ , $\theta_{Knee}$ , $\theta_{Ankle}$ , and $L^{M}$ were randomly initiated within the range of 0.60≤Erectness≤1 and every variable threshold. In addition, the search direction was randomly selected on the foundation of the gradient descent method; this process was repeated 3,536 times. The largest Object among these 3,536 sets of numerical results was approximated as the global optimal solution. During the course, other sets of optimization results were also weighted as simulation experiments of bipedal standing in chimps.

Probing deeper into the relationship between each pair of the biomechanical parameters, the Pearson correlation analysis of the outcomes from simulating experiments was accomplished.

## 3 Results

In this study, the effects of skeletal architecture and muscle properties on bipedal standing in chimps were investigated using modeling and simulation. The global optimal solution of Object was achieved from 3,536 sets of optimization results to ascertain the optimal posture for bipedal standing in chimps. It is worth noting that these 3,536 sets of numerical results can also be considered as 3,536 simulations of bipedal standing experiments. Based on these simulations, the numerical relationships among Object , Erectness , and Fatigue can be analyzed, and the biomechanical relationships among the hip, knee, and ankle joint angles; muscle activation; muscle lengths; and muscle forces of the hind limbs can be further analyzed.

### 3.1 Numerical trade-offs between erectness and fatigue

Taking Object as the objective function, simultaneously maximizing Erectness and minimizing Fatigue , numerical optimization was conducted 3,536 times, and 3,536 randomized simulations of bipedal standing experiments in chimps were performed.

**FIGURE 3:**
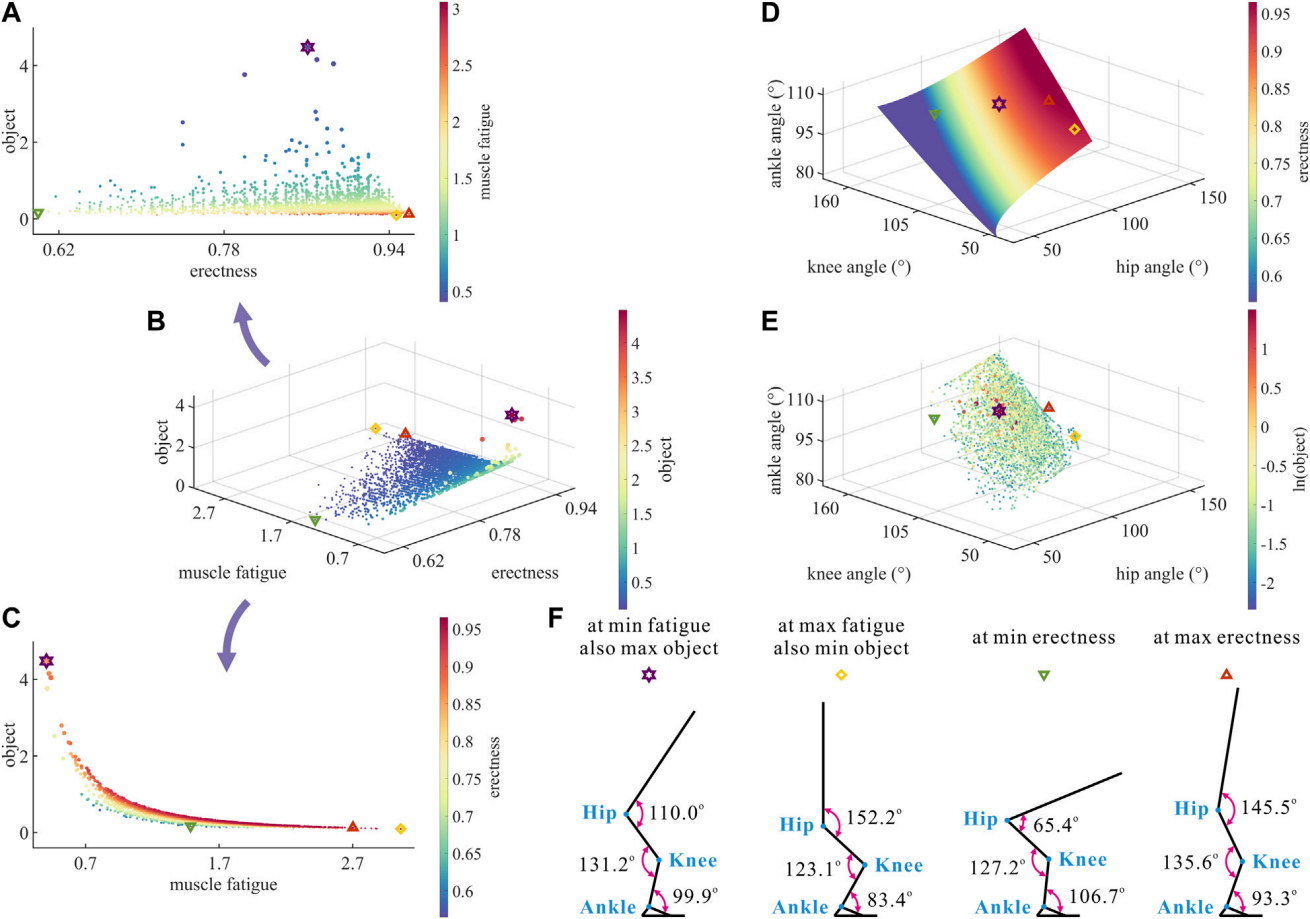
**(A–C)** Results of optimization objective, standing erectness, and muscle fatigue of the lower limb in the 3,536 simulation experiments of bipedal standing. Marked points: purple hexagram: both the optimal and easiest BSP; yellow diamond: both the worst and hardest BSP; green downward-pointing triangle: lowest BSP; red upward-pointing triangle: highest BSP. **(D)** Domain of the hip, knee, and ankle joint angles. **(E)** Results of the hip, knee, and ankle joint angles in the 3,536 simulation experiments of bipedal standing. **(F)** Joint angles, respectively, at (purple hexagram) both the optimal and easiest BSP, (yellow diamond) both the worst and hardest BSP, (green downward-pointing triangle) lowest BSP, and (red upward-pointing triangle) highest BSP.

As shown in Figures 3A–C, the same degree of Erectness may correspond to different degrees of Fatigue , and in turn, the same degree of Fatigue may correspond to different degrees of Erectness . As the value of Object gradually increased from a minimum of 0.096 to a maximum of 4.480, the degree of Fatigue generally decreased from a maximum of 3.060 to a minimum of 0.407, whereas the degree of Erectness did not show a clear pattern of change. When Object reached a maximum of 4.480, which is the optimal BSP, although Fatigue also reached a minimum of 0.407 (the easiest BSP), Erectness reached neither the minimum of 0.600 nor the maximum of 0.959, but 0.861. This indicated that Fatigue is higher than 0.407 when Erectness is either less than or more than 0.861, so that Object is reduced. Moreover, when Fatigue reached a maximum of 3.060 (the hardest BSP), Erectness was 0.947, which is not significantly different from its maximum of 0.959. Additionally, the degree of Fatigue was higher at the maximum value of Erectness (the highest BSP) than at the minimum value of Erectness (the lowest BSP), which were 2.702 and 1.486, respectively. This result is rather counterintuitive, indicating that too high a degree of Erectness would not lead to a decrease in Fatigue . These results contrast with those for humans, where the optimal BSP corresponds to both the highest Erectness and the lowest Fatigue (Pontzer et al., 2009), with Erectness and Fatigue being almost negatively correlated (Neumann, 2013).

As a result, there is a numerical trade-off between the degree of Erectness and the degree of Fatigue in the quest of the optimal BSP for chimps.

Although Object is the ratio of Erectness to Fatigue , the latter two are not completely independent variables. The degree of Erectness is directly dependent on $\theta_{Hip}$ , $\theta_{Knee}$ , and $\theta_{Ankle}$ . The degree of Fatigue is directly dependent on the muscle activation of hind limb MTUs ${act}_{i}$ ( i=GM,bflh,semimem,semiten,Vas,RF,Gasl,Gasm,Sol,TA ), which indirectly depends on $L^{MTU}$ and $L^{M}$ (see Section 2.1.2 for details). Because $L^{MTU}$ depends directly on $\theta_{Hip}$ , $\theta_{Knee}$ , and $\theta_{Ankle}$ , Fatigue is indirectly affected by $\theta_{Hip}$ , $\theta_{Knee}$ , $\theta_{Ankle}$ , and $L_{i}^{M}$ ( i=GM,bflh,semimem,semiten,Vas,RF,Gasl,Gasm,Sol,TA ).

In summary, the numerical trade-off between the degree of Erectness and the degree of Fatigue requires a comprehensive consideration of $\theta_{Hip}$ , $\theta_{Knee}$ , $\theta_{Ankle}$ , act , and $L^{M}$ .

### 3.2 Numerical trade-offs among hip–knee–ankle angles by skeletal architecture

As shown in Figure 3D, the degree of Erectness increased with $\theta_{Hip}$ , $\theta_{Knee}$ , and $\theta_{Ankle}$ , wherein the change caused by $\theta_{Hip}$ was the most evident. This is attributable to the fact that the hip, knee, and ankle joints, respectively, drive the HAT, thigh, and shank segments, among which the HAT segment is the longest.

As shown in Figure 3E, the motion range of $\theta_{Hip}$ was 105.59±48.49° , the motion range of $\theta_{Knee}$ was 118.07±43.83° , and the motion range of $\theta_{Ankle}$ was 95.42±17.05° in 3,536 simulation experiments of bipedal standing in chimps.

As shown in Figure 3F, the corresponding $\theta_{Hip}$ , $\theta_{Knee}$ , and $\theta_{Ankle}$ of the optimal BSP and easiest BSP were, respectively, 110.02° , 131.19° , and 99.92° , which is consistent with the existing measurement data of the middle stance during bipedal walking in chimps (Jenkins, 1972; O'Neill et al., 2015).

Taking the optimal BSP as the benchmark, the lowest BSP showed a significant decrease in $\theta_{Hip}$ ( −40.60% ) and insignificant changes in $\theta_{Knee}$ ( −3.07% ) and $\theta_{Ankle}$ ( +6.80% ), while the highest BSP showed a significant increase in $\theta_{Hip}$ ( +32.24% ) and insignificant changes in $\theta_{Knee}$ ( +3.36% ) and $\theta_{Ankle}$ ( −6.59% ). This is not only consistent with the pattern shown in Figure 3D, but also suggests that $\theta_{Hip}$ , $\theta_{Knee}$ , and $\theta_{Ankle}$ cannot be increased or decreased at the same time compared to the optimal BSP in the effort to maintain balance in chimps.

It is noteworthy that the maximum degree of Fatigue (the hardest BSP) did not correspond to the highest or lowest BSP, whereas $\theta_{Hip}$ increased significantly ( +38.32% ), $\theta_{Knee}$ decreased slightly ( −6.18% ), and $\theta_{Ankle}$ decreased significantly ( −16.55% ) compared to the optimal BSP. This signifies that simultaneous changes in $\theta_{Hip}$ and $\theta_{Ankle}$ cause a greater degree of Fatigue than simultaneous changes in $\theta_{Hip}$ and $\theta_{Knee}$ .

Therefore, there is a numerical trade-off between the hip–knee–ankle joint angles in the quest of the optimal BSP for chimps.

The hip–knee–ankle joint angles directly decided $L^{MTU}$ of the hind limbs, thereby influencing the range of values for $L^{M}$ , which, in turn, indirectly influenced the value of the degree of Fatigue .

For uni-articular MTUs, the larger the $\theta_{Hip}$ was, the smaller the $L^{MTU}$ of the extensor GM; the larger the $\theta_{Knee}$ was , the smaller the $L^{MTU}$ of the extensor Vas; the larger the $\theta_{Ankle}$ was, the smaller the $L^{MTU}$ of the extensor Sol and the larger the $L^{MTU}$ of the flexor TA.

For bi-articular MTUs, bflh, semimem, and semiten were both hip extensors and knee flexors, RF was both the hip flexor and knee extensor, and gasl and gasm were both knee flexors and ankle extensors. The variation in the $L^{MTU}$ in bi-articular MTUs depended on the specific magnitude of the angular variations.

As shown in Figures 5–7, as long as $\theta_{Hip}$ , $\theta_{Knee}$ , $\theta_{Ankle}$ , and $L_{i}^{M}$ ( i=GM,bflh,semimem,semiten,Vas,RF,Gasl,Gasm,Sol,TA ) were settled, ${act}_{i}$ ( i=GM,bflh,semimem,semiten,Vas,RF,Gasl,Gasm,Sol,TA ) could be uniquely certified. Ergo, Fatigue can be derived.

To summarize, the numerical trade-offs between $\theta_{Hip}$ , $\theta_{Knee}$ , and $\theta_{Ankle}$ also demand the contemplation of muscle activation of lower-limb MTUs.

### 3.3 Numerical trade-offs among muscle parameters of MTUs by muscle properties

**FIGURE 4:**
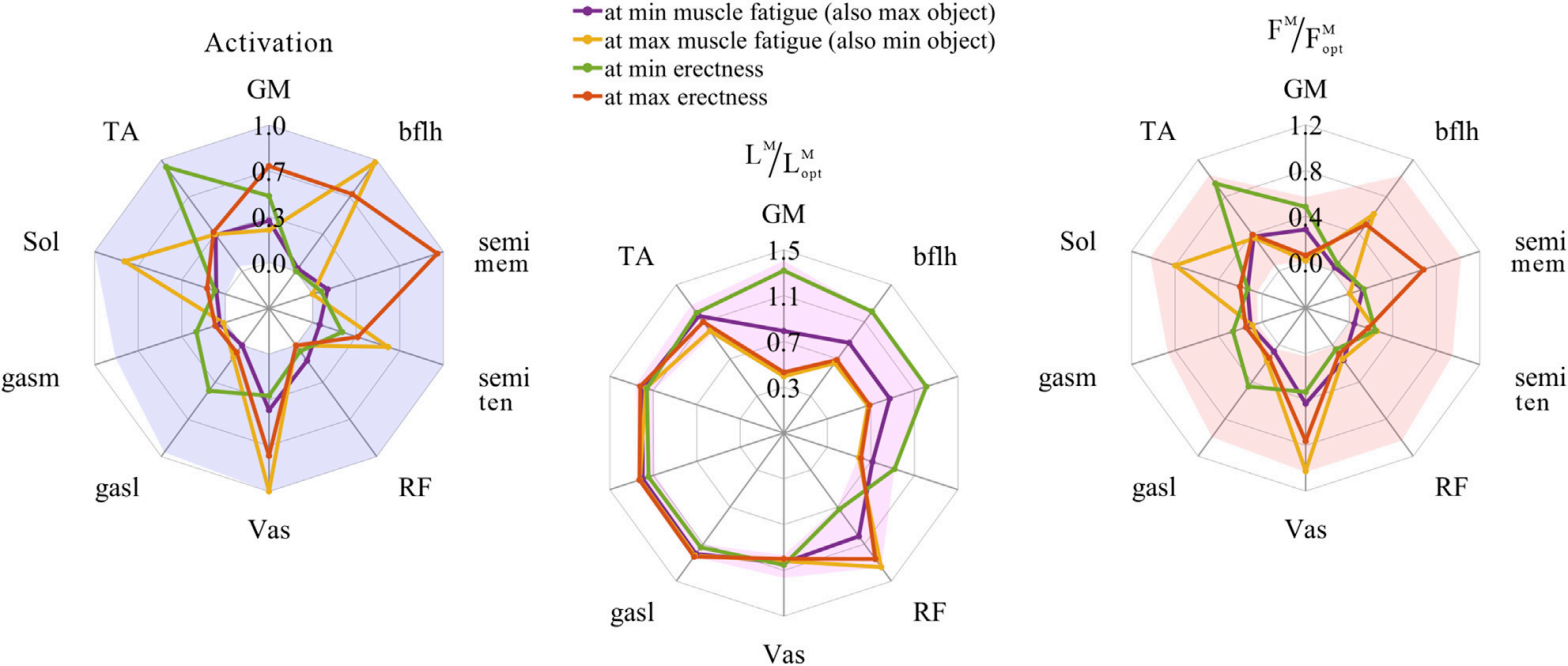
Distribution and value ranges of activation, relative muscle lengths, and relative muscle forces of the lower-limb MTUs. Marked lines: purple: both the optimal and easiest BSP; yellow: both the worst and hardest BSP; green: lowest BSP; red: highest BSP.

The distribution and value ranges of the activation, relative muscle lengths, and relative muscle forces of the lower-limb MTUs in the 3,536 experimental simulations are shown in Figure 4.

#### 3.3.1 Relative muscle lengths

According to Section 2.1.2, the degree of muscle activation in the lower-limb MTUs directly depends on the relative muscle lengths.

**FIGURE 5:**
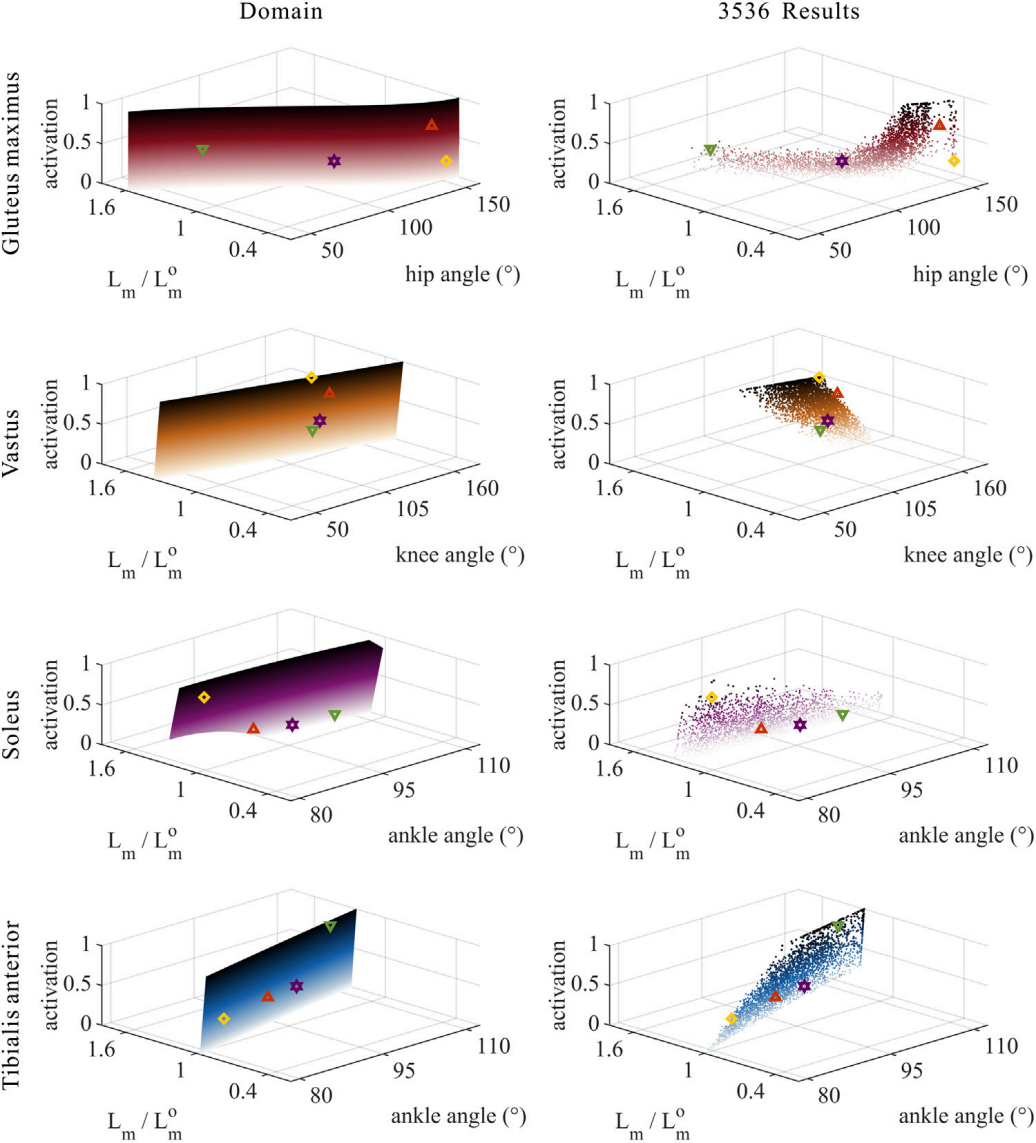
Domains (surfaces) and results in the 3,536 simulation experiments (scatters) of activation, relative muscle lengths, and joint angles for the uni-articular MTUs (gluteus maximus, vastus, soleus, and tibialis anterior). Marked points: purple hexagram: both the optimal and easiest BSP; yellow diamond: both the worst and hardest BSP; green downward-pointing triangle: lowest BSP; red upward-pointing triangle: highest BSP.

As shown in Figures 5, 8, 9, for uni-articular MTUs, there was a correspondence between the relative muscle lengths and joint angles. For the hip extensor GM, $\frac{l_{m}^{GM}}{l_{opt}^{GM}}$ was negatively correlated with $\theta_{Hip}$ ( r=−1.00 , p<0.05 ). For the knee extensor Vas, $\frac{l_{m}^{Vas}}{l_{opt}^{Vas}}$ was negatively correlated with $\theta_{Knee}$ ( r=−0.95 , p<0.05 ). For the ankle extensor Sol, $\frac{l_{m}^{Sol}}{l_{opt}^{Sol}}$ was negatively correlated with $\theta_{Ankle}$ ( r=−0.78 , p<0.05 ). For the ankle flexor TA, $\frac{l_{m}^{TA}}{l_{opt}^{TA}}$ was positively correlated with $\theta_{Ankle}$ ( r=0.99 , p<0.05 ).

**FIGURE 6:**
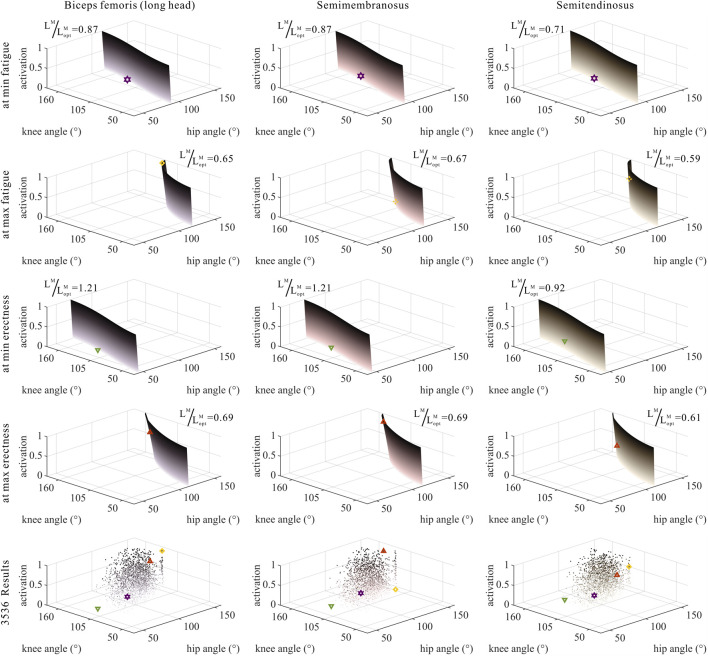
Domains (surfaces) and results in the 3,536 simulation experiments (scatters) of activation, relative muscle lengths, and joint angles for bi-articular MTUs [biceps femoris (long head), semimembranosus, and semitendinosus]. Marked points: purple hexagram: both the optimal and easiest BSP; yellow diamond: both the worst and hardest BSP; green downward-pointing triangle: lowest BSP; red upward-pointing triangle: highest BSP.

**FIGURE 7:**
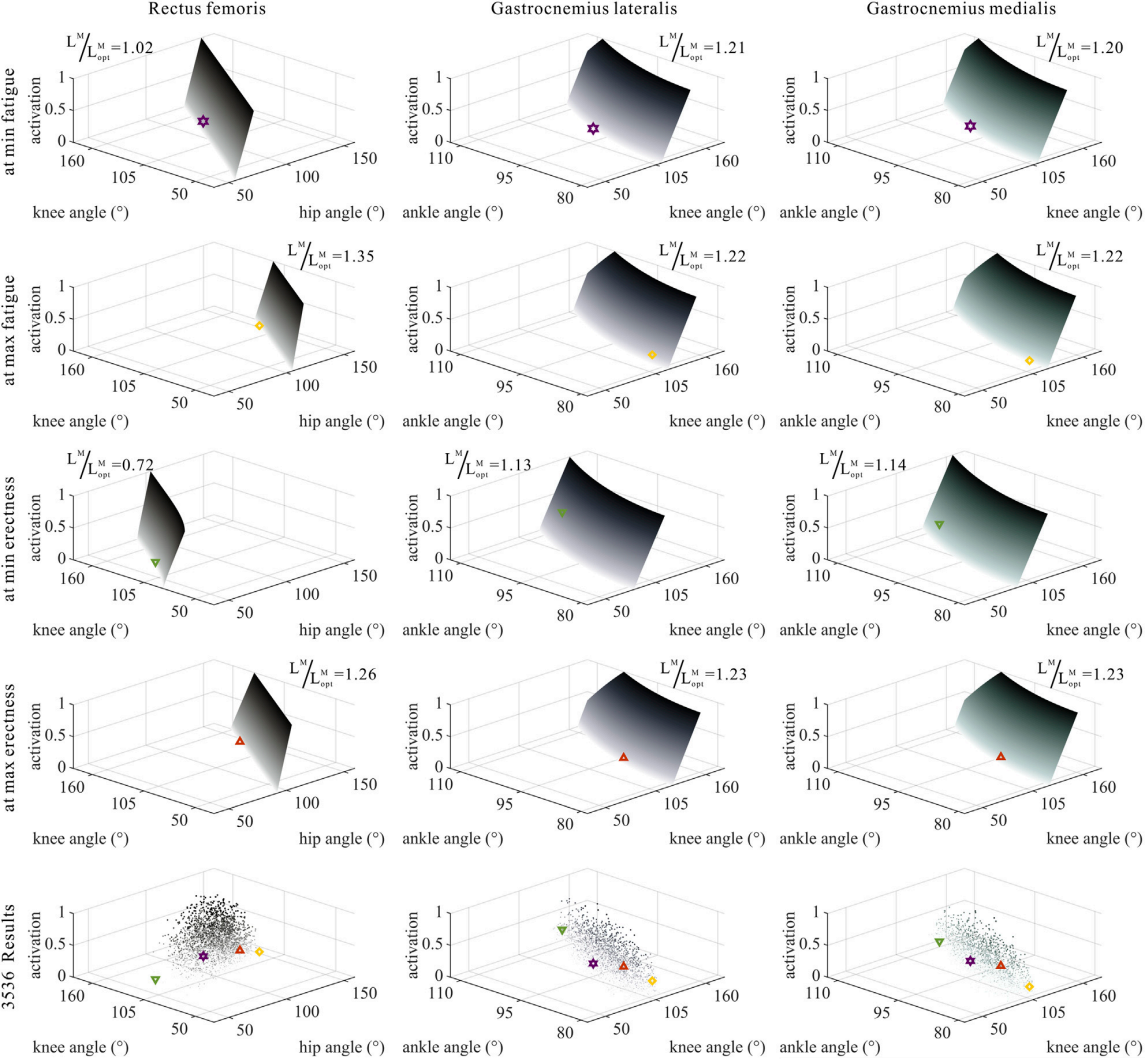
Domains (surfaces) and results in the 3,536 simulation experiments (scatters) of activation, relative muscle lengths, and joint angles for bi-articular MTUs (rectus femoris, gastrocnemius lateralis, and gastrocnemius medialis). Marked points: purple hexagram: both the optimal and easiest BSP; yellow diamond: both the worst and hardest BSP; green downward-pointing triangle: lowest BSP; red upward-pointing triangle: highest BSP.

As shown in Figures 6, 7, 10, 11, and 12, for bi-articular MTUs, the correspondence between the relative muscle lengths and joint angles still existed but the degree varied among joints. For bflh, semimem, and semiten, which are both hip extensors and knee flexors, the relative muscle lengths were negatively correlated with $\theta_{Hip}$ ( $\frac{l_{m}^{bflh}}{l_{opt}^{bflh}}$ : r=−0.98 , p<0.05 ; $\frac{l_{m}^{semimem}}{l_{opt}^{semimem}}$ : r=−0.98 , p<0.05 ; $\frac{l_{m}^{semiten}}{l_{opt}^{semiten}}$ : r=−0.97 , p<0.05 ) and less correlated with $\theta_{Knee}$ ( $\frac{l_{m}^{bflh}}{l_{opt}^{bflh}}$ : r=−0.31 , p<0.05 ; $\frac{l_{m}^{semimem}}{l_{opt}^{semimem}}$ : r=−0.30 , p<0.05 ; $\frac{l_{m}^{semiten}}{l_{opt}^{semiten}}$ : r=−0.30 , p<0.05 ). For RF, both the hip flexor and knee extensor, the relative muscle length was positively correlated with $\theta_{Hip}$ ( $\frac{l_{m}^{RF}}{l_{opt}^{RF}}$ : r=0.90 , p<0.05 ) and less correlated with $\theta_{Knee}$ ( $\frac{l_{m}^{RF}}{l_{opt}^{RF}}$ : r=0.07 , p<0.05 ). For gasl and gasm, which are both knee flexors and ankle extensors, the relative muscle lengths were positively correlated with $\theta_{Knee}$ ( $\frac{l_{m}^{Gasl}}{l_{opt}^{Gasl}}$ : r=0.57 , p<0.05 ; $\frac{l_{m}^{Gasm}}{l_{opt}^{Gasm}}$ : r=0.59 , p<0.05 ) and less correlated with $\theta_{Ankle}$ ( $\frac{l_{m}^{Gasl}}{l_{opt}^{Gasl}}$ : r=0.20 , p<0.05 ; $\frac{l_{m}^{Gasm}}{l_{opt}^{Gasm}}$ : r=0.21 , p<0.05 ).

#### 3.3.2 Muscle activation

As shown in Figures 5, 9, for uni-articular MTUs, there was a correspondence between the muscle activation and joint angles. For the hip extensor GM, ${act}^{GM}$ was positively correlated with $\theta_{Hip}$ ( r=0.56 , p<0.05 ). For the knee extensor Vas, ${act}^{Vas}$ was negatively correlated with $\theta_{Knee}$ ( r=−0.80 , p<0.05 ). For the ankle extensor Sol, ${act}^{Sol}$ was negatively correlated with $\theta_{Ankle}$ ( r=−0.40 , p<0.05 ). For the ankle flexor TA, ${act}^{TA}$ was positively correlated with $\theta_{Ankle}$ ( r=0.66 , p<0.05 ). Among them, GM did not satisfy the rule that muscle activation is negatively correlated with joint angles for extensors and positively correlated with joint angles for flexors, which will be discussed in detail in Section 4.

As shown in Figures 6, 7, 12, for bi-articular MTUs, the correspondence between the muscle activation and joint angles still existed in some but the degree was much lower. For bflh, semimem, and semiten, which are both hip extensors and knee flexors, the muscle activation was positively correlated with $\theta_{Hip}$ ( ${act}^{bflh}$ : r=0.20 , p<0.05 ; ${act}^{semimem}$ : r=0.14 , p<0.05 ; ${act}^{semiten}$ : r=0.09 , p<0.05 ) and also positively correlated with $\theta_{Knee}$ ( ${act}^{bflh}$ : r=0.28 , p<0.05 ; ${act}^{semimem}$ : r=0.26 , p<0.05 ; ${act}^{semiten}$ : r=0.18 , p<0.05 ). For RF, both the hip flexor and knee extensor, the muscle activation was negatively correlated with $\theta_{Hip}$ ( ${act}^{RF}$ : r=−0.29 , p<0.05 ) and also negatively correlated with $\theta_{Knee}$ ( ${act}^{RF}$ : r=−0.22 , p<0.05 ). For gasl and gasm, which are both knee flexors and ankle extensors, the correlation was neglectable.

These results suggest that the corresponding relationship between muscle activation and joint angles in bi-articular MTUs does not match that in uni-articular MTUs. It was speculated that bi-articular MTUs play a paramount role in regulating balance during bipedal standing in chimps.

#### 3.3.3 Relative muscle forces

**FIGURE 8:**
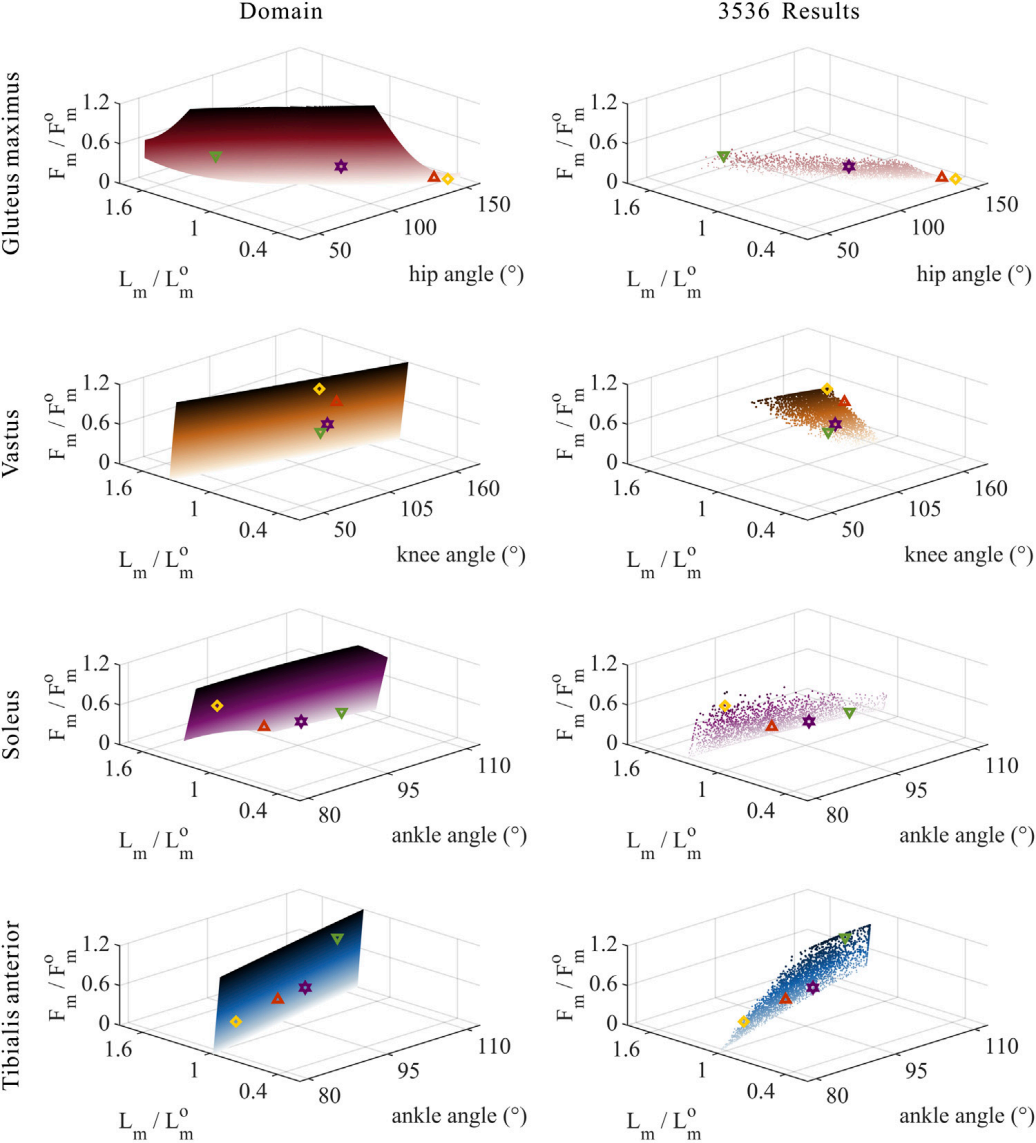
Domains (surfaces) and results in the 3,536 simulation experiments (scatters) of relative muscle forces, relative muscle lengths, and joint angles for uni-articular MTUs (gluteus maximus, vastus, soleus, and tibialis anterior). Marked points: purple hexagram: both the optimal and easiest BSP; yellow diamond: both the worst and hardest BSP; green downward-pointing triangle: lowest BSP; red upward-pointing triangle: highest BSP.

**FIGURE 9:**
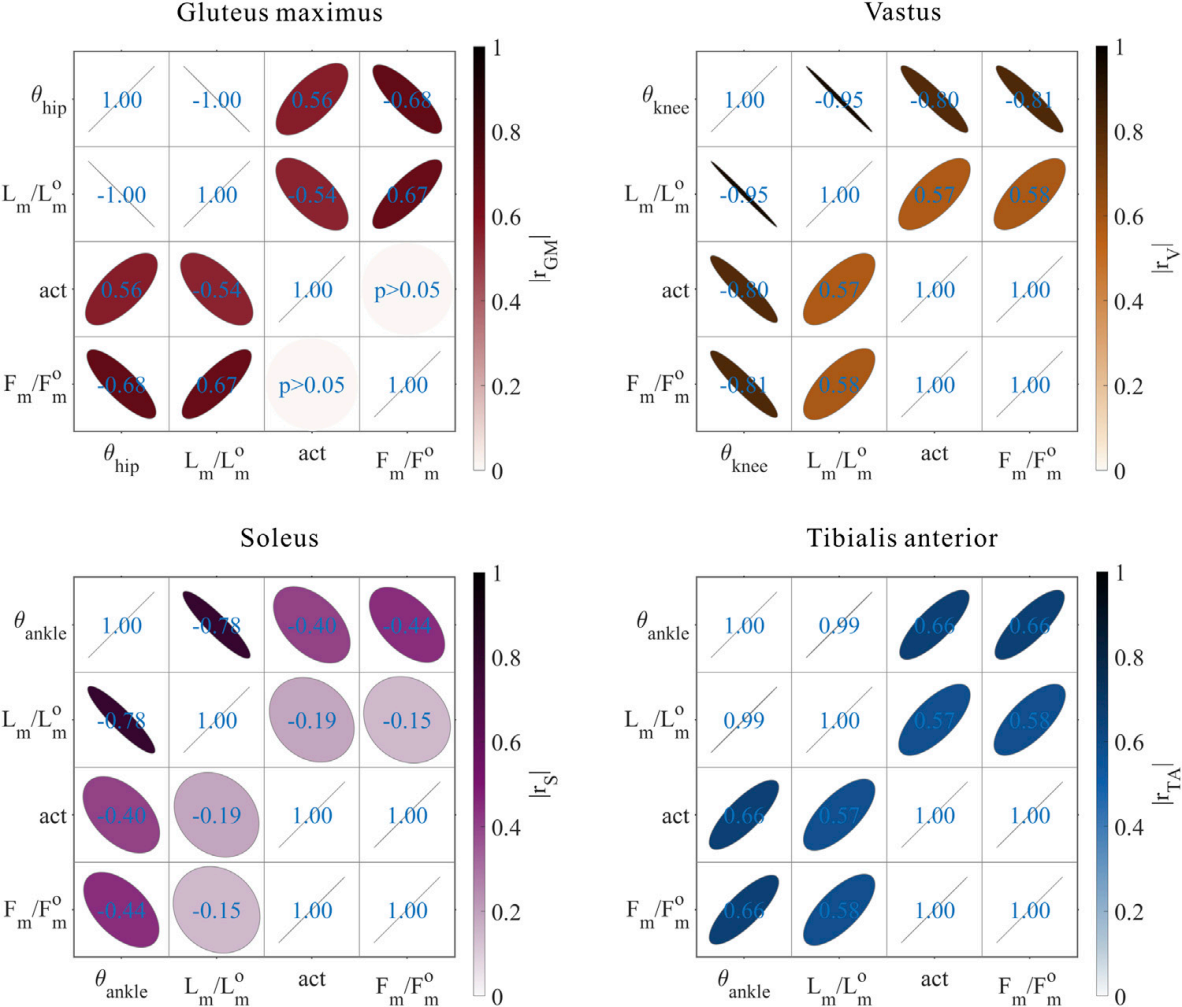
Results of the correlation analysis of joint angles, relative muscle lengths, activation, and relative muscle forces among the 3,536 simulation experiments for the uni-articular MTUs (gluteus maximus, vastus, soleus, and tibialis anterior).

As shown in Figures 8, 9, for uni-articular MTUs, there was a correspondence between the relative muscle forces and joint angles. For the hip extensor GM, $\frac{F_{m}^{GM}}{F_{opt}^{GM}}$ was negatively correlated with $\theta_{Hip}$ ( r=−0.68 , p<0.05 ). For the knee extensor Vas, $\frac{F_{m}^{Vas}}{F_{opt}^{Vas}}$ was negatively correlated with $\theta_{Knee}$ ( r=−0.81 , p<0.05 ). For the ankle extensor Sol, $\frac{F_{m}^{Sol}}{F_{opt}^{Sol}}$ was negatively correlated with $\theta_{Ankle}$ ( r=−0.44 , p<0.05 ). For the ankle flexor TA, $\frac{F_{m}^{TA}}{F_{opt}^{TA}}$ was positively correlated with $\theta_{Ankle}$ ( r=0.66 , p<0.05 ).

These results satisfied the rule that relative muscle forces are negatively correlated with joint angles for extensors and positively correlated with those for flexors.

**FIGURE 10:**
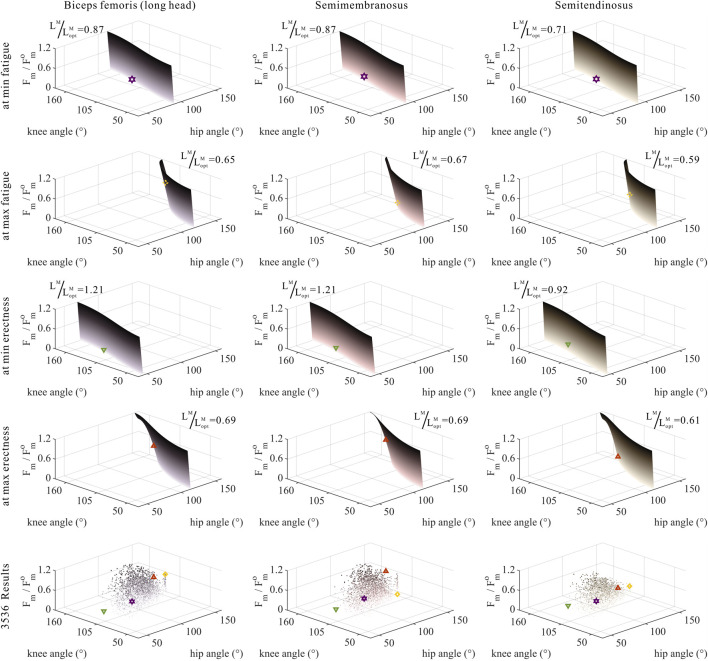
Domains (surfaces) and results in the 3,536 simulation experiments (scatters) of relative muscle forces, relative muscle lengths, and joint angles for bi-articular MTUs [biceps femoris (long head), semimembranosus, and semitendinosus]. Marked points: purple hexagram: both the optimal and easiest BSP; yellow diamond: both the worst and hardest BSP; green downward-pointing triangle: lowest BSP; red upward-pointing triangle: highest BSP.

**FIGURE 11:**
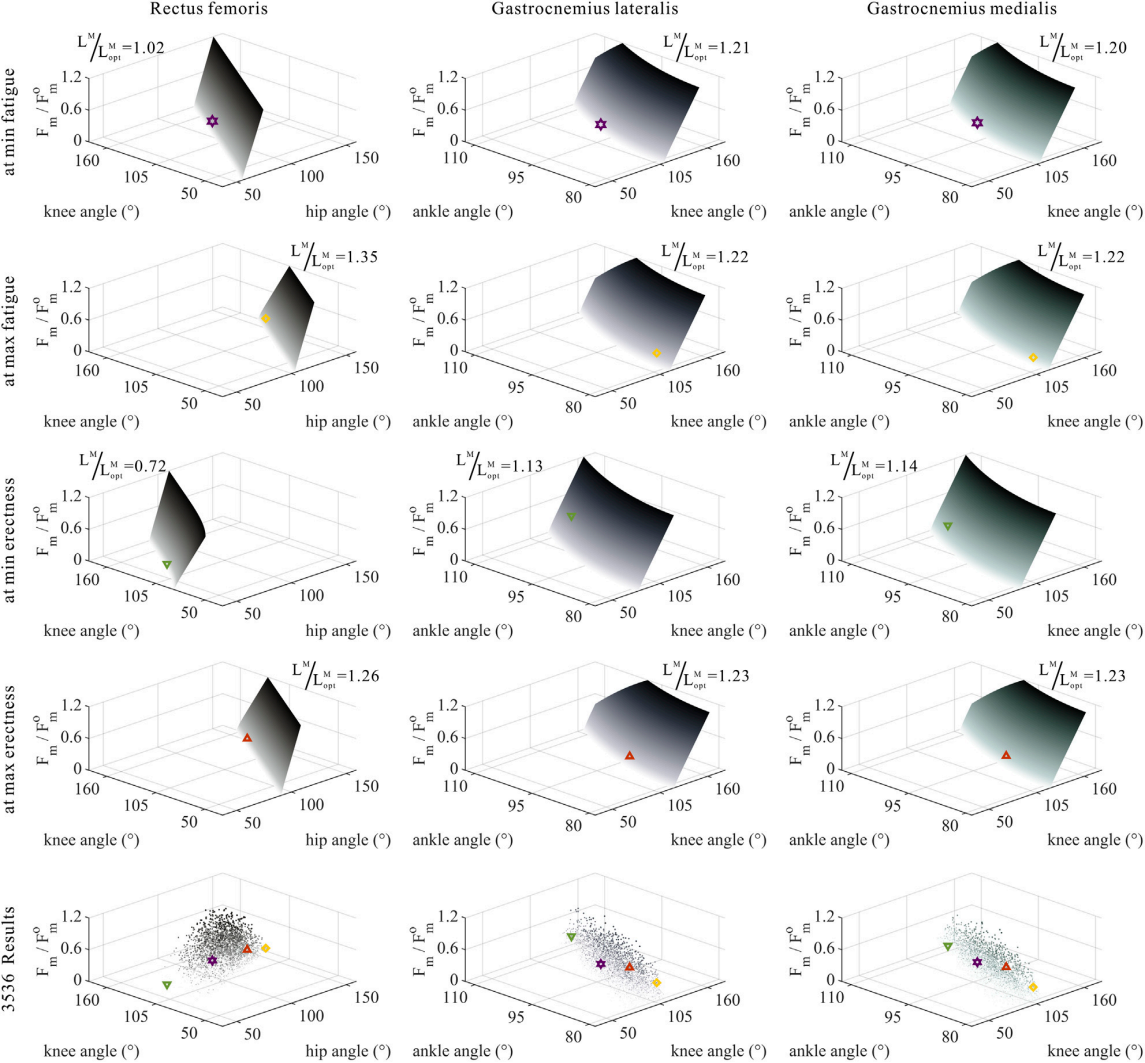
Domains (surfaces) and results in the 3,536 simulation experiments (scatters) of relative muscle forces, relative muscle lengths, and joint angles for bi-articular MTUs (rectus femoris, gastrocnemius lateralis, and gastrocnemius medialis). Marked points: purple hexagram: both the optimal and easiest BSP; yellow diamond: both the worst and hardest BSP; green downward-pointing triangle: lowest BSP; red upward-pointing triangle: highest BSP.

**FIGURE 12:**
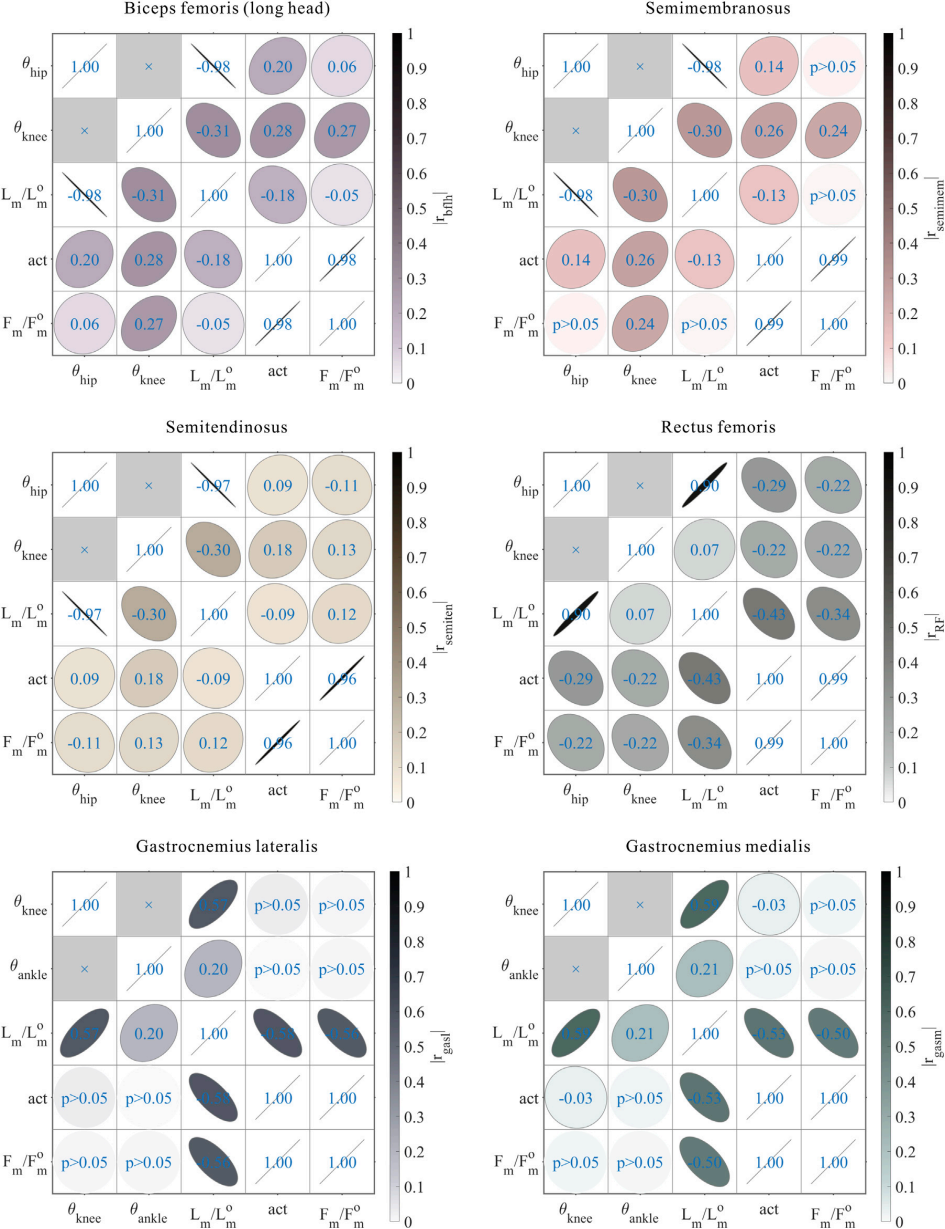
Results of the correlation analysis of joint angles, relative muscle lengths, activation, and relative muscle forces among the 3,536 simulation experiments for the bi-articular MTUs [biceps femoris (long head), semimembranosus, semitendinosus, rectus femoris, gastrocnemius lateralis, and gastrocnemius medialis].

As shown in Figures 10, 11, 12, for bi-articular MTUs, the correspondence between the relative muscle forces and joint angles was too weak to form a pattern. These results indicate that the relative muscle forces of bi-articular MTUs cannot be conjectured directly from $\theta_{Hip}$ , $\theta_{Knee}$ , and $\theta_{Ankle}$ , which likewise took the next step in validating that bi-articular MTUs play a fundamental role in regulating balance during bipedal standing in chimps.

## 4 Discussion

Our study substantiated the premise that when the common chimpanzee is bipedal standing, 1) it cannot simultaneously achieve the maximum Erectness and the minimum Fatigue , and excessive Erectness would not lead to the reduction of Fatigue ; 2) the hip–knee–ankle joint angles corresponding to the optimal BSP are consistent with the measurement data (Jenkins, 1972; O'Neill et al., 2015) of the middle stance during bipedal walking of chimps; 3) for uni-articular MTUs, the relationship between the muscle activation and the corresponding joint angle, that between the relative muscle lengths and the corresponding joint angle, together with that between the relative muscle forces and the corresponding joint angle is, generally negatively correlated for extensors and positively correlated for flexors; and 4) for bi-articular MTUs, the relationship between the relative muscle lengths and the corresponding joint angles is still negatively correlated for extensors and positively correlated for flexors, but that between the muscle activation and the corresponding joint angles, coupled with the relationship between the relative muscle forces and the corresponding joint angles, is hardly correlated.

### 4.1 Biomechanical effects of skeletal architecture on the bipedal standing posture of chimps

The lumbar lordosis is absent in chimps; their almost-rigid lumbar spine restrains further extension of the HAT, which compels the lower-limb MTUs to bear greater lumbar-bending stresses during bipedal standing (Lovejoy et al., 2009). In this study, the pelvis was included in the HAT segment, and not as a separate segment, when we established the musculoskeletal model, ignoring the degree of freedom between the lumbar spine and pelvis.

The lower limbs of chimps are evidently shorter than the slender legs of humans (Schultz, 1937; Young et al., 2010); evidently, their HAT segment is longer than the thigh and shank segments. The results presented in Section 3.1 unmasked that this very skeletal architecture leads to the variation in the degree of Erectness , which was induced mainly by the hip joint angle.

The elongated and laterally oriented ischia of chimps limit the range of motion of the hip joint. When the hip joint is extended, the moment arms of the GM and hamstrings (bflh, semimem, and semiten), together with the length of the GM, rapidly decrease (Robinson, 1972; McHenry, 1975; Kozma et al., 2018). The results obtained in Section 3.3.1 suggest that this very skeletal architecture leads to the relative muscle lengths of bi-articular MTUs that span the hip and knee joints, namely, the bflh, semimem, semiten, and RF, to be more susceptible to the hip joint angle.

The elongated and dorsally oriented ilia of chimps entail that only the movement of the gluteus maximus ischiofemoralis is regulated in the sagittal plane, whereas the movement of the gluteus maximus poprius is mainly curbed in the coronal plane (Stern, 1972; Tuttle et al., 1979; Lieberman et al., 2006). The results obtained in Section 3.3.2 indicate that this skeletal architecture leads to a positive correlation between the muscle activation of GM, uni-articular extensor, and hip joint angle, in contrast to the pattern satisfied by other uni-articular MTUs, where muscle activation was negatively correlated with joint angles for extensors and positively correlated with joint angles for flexors.

### 4.2 Biomechanical effects of muscle properties on the bipedal standing posture of chimps

The structure of the MTU governs its muscle–tendon length distribution and its ability to produce force (Biewener and Roberts, 2000; Charles et al., 2022). For instance, the MTU with a shorter muscle and longer tendon is designed to generate more economic force, and that with a longer muscle and shorter tendon is suitable for maintaining the stability of joints. The skeletal muscles of chimps have, on average, longer muscle fibers (Isler, 2005); thus, the maximum dynamic force output in muscles of the same size is 1.35 times greater than that of humans (O’Neill et al., 2017). The results presented in Section 3.3.2 and Section 3.3.3 suggest that this muscle property leads the muscle activation and relative muscle forces of bi-articular MTUs to the violation of the pattern conformed by uni-articular MTUs, where these parameters were negatively correlated with joint angles for extensors and positively correlated with joint angles for flexors. It was meditated that during the bipedal standing of chimps, bi-articular MTUs mainly played a role in harmonizing balance, owing to the longer muscle fibers than those of uni-articular MTUs.

The gluteus maximus of chimps originates from the sacro-iliac region, coccyx, sacrotuberous ligament, and ischial tuberosity, while it inserts in the vastus lateralis aponeurosis (a part of the iliotibial tract) and along the lateral side of the femoral diaphysis (Stern, 1972). Therefore, compared with humans, the gluteus maximus of chimps faces more laterally and then acts more in the coronal plane than that in the sagittal plane (Lovejoy et al., 2002). The results of Section 3.3.2 indicated that this muscle property leads to a positive correlation between the muscle activation and the hip joint angle for the uni-articular extensor GM, in contrast to the pattern met by other uni-articular MTUs where muscle activation was negatively correlated with joint angles for extensors and positively correlated with joint angles for flexors.

The gluteus maximus of chimps is considerably smaller than that of humans, deteriorating the complementary function of the hamstrings in extending the hip joint (Stern and Susman, 1981; Lieberman et al., 2006). The results presented in Section 3.3.2 and Section 3.3.3 indicate that this muscle property leads to a further increase in both the muscle activation and muscle forces of the hamstrings when the hip joint is extended.

Unlike humans, chimps lack the external Achilles tendon in the triceps surae (gasl, gasm, and Sol), the PCSAs of which are relatively small. Therefore, the force production of all these MTUs is small within the motion range of the ankle joint (Thorpe et al., 1999; Payne et al., 2006b).

### 4.3 Limitations and practical implications

Due to the incompleteness of anatomical information from a single specimen of the common chimpanzee, multiple different specimens were used to build the musculoskeletal model. Therefore, parameters of the skeletal architecture and muscle properties were scaled based on the principle of geometric similarity, which might not be an appropriate assumption. Muscle force-generating capacities of mammals in general were found to be proportional to the body mass raised to the power of 0.8 (Alexander et al., 1981) and to differ in divergent muscles. However, research studies also suggested that while peak isometric muscle forces calculated by the scaling method significantly differed from those measured, the gleaned muscle-force functions were quite similar (Scovil et al., 2006; Redl et al., 2007; Correa & Pandy, 2011). This indication supports the application of mass–length scaling in the musculoskeletal model development.

Though mainly lower-limb MTUs were considered in the musculoskeletal model, core muscles, such as multifidus, also play a critical role in the bipedalism (Wang et al., 2023). Research studies implied that multifidus controls trunk movement primarily in the sagittal plane during the bipedal and quadrupedal movements in chimps (Shapiro & Jungers, 1988; Shapiro & Jungers, 1994). Consequently, multifidus is worth being used in the model, only if the anatomical data are available.

The musculoskeletal model proposed in this paper is able to predict how changes in the skeletal architecture and muscle properties could alter the force-generating capacity of MTUs. This will enhance the understanding of causal relationships between the musculoskeletal system and locomotor characteristics in primates and advance the comprehension of bipedal evolution in humans.

The biomechanical limitations of the common chimpanzee were elucidated in this paper, which inspire the design of prosthetic devices and assistive technologies for people with impaired mobility, and of robotic systems that better mimic the movements of humans.

## 5 Conclusion

In this study, to explore the effects of skeletal architecture and muscle properties on bipedal standing in chimps from the perspective of biomechanics, we established a whole-body musculoskeletal model of the common chimpanzee and developed experimental simulations of bipedal standing. Chimps bipedally stand in a “bent-hip, bent-knee” posture due to their skeletal architecture, such as the almost rigid lumbar spine, relatively long HAT segment, elongated and laterally oriented ischia, and elongated and dorsally oriented ilia. The relationship between muscle activation, relative muscle lengths, together with relative muscle forces, and the corresponding joint angle varies between uni-articular and bi-articular MTUs because of muscle properties, such as the muscle–tendon length distribution, insertion, and shape. It would appear that bi-articular MTUs chiefly contribute to balance. Future research could continue to complete the anatomical dataset of chimps, refine the relationships between the musculoskeletal system and locomotor characteristics in primates, and even design wearable equipment or bipedal robotics based on the drawn mechanism.

## Data Availability

The original contributions presented in the study are included in the article/Supplementary Material; further inquiries can be directed to the corresponding author.
