# Supplementary material for: Exploring the effects of skeletal architecture and muscle properties on bipedal standing in the common chimpanzee (Pan troglodytes) from the perspective of biomechanics
Source: Front Bioeng Biotechnol. 2023 May 5;11:1140262. doi: 10.3389/fbioe.2023.1140262 (PMC10196953; doi:10.3389/fbioe.2023.1140262)
Supplement: Supplementary file 1 [file DataSheet1.pdf]

Supplementary Material

Exploring the effects of skeletal architecture and muscle properties on bipedal standing in the common chimpanzee (Pan troglodytes) from the perspective of biomechanics

Xiao-Wei Xv, Wen-Bin Chen*, Cai-Hua Xiong*, Bo Huang, Long-Fei Cheng and Bai-Yang Sun

*** Correspondence:** Cai-Hua Xiong: chxiong@hust.edu.cn

Wen-Bin Chen: wbchen@hust.edu.cn

1 Supplementary Tables

Table S1. Segmental parameters of the musculoskeletal model.

Parameter		Description	Value	Source
L_{total} **(mm)**		total body length	1213.3	Thorpe et al. (1999)<br>Isler et al. (2006)
m_{total} **(kg)**		total body mass	37	Thorpe et al. (1999)
**HAT**	L_{HAT} **(mm)**	length of HAT	633.3	Isler et al. (2006)<br>Thorpe et al. (1999)
	m_{HAT} **(kg)**	mass of HAT	28.228	Isler et al. (2006)<br>Thorpe et al. (1999)
	D_{HAT}^{CoM} **(mm)**	distance between HAT CoM and hip joint	217.4	Isler et al. (2006)<br>Thorpe et al. (1999)
**One Thigh**	L_{Thigh} **(mm)**	length of thigh	290	Thorpe et al. (1999)
	m_{Thigh} **(kg)**	mass of one thigh	2.584	Isler et al. (2006)<br>Thorpe et al. (1999)
	D_{Thigh}^{CoM} **(mm)**	distance between thigh CoM and knee joint	163.0	Isler et al. (2006)<br>Thorpe et al. (1999)
	L_{femur} **(mm)**	length of femur	290	Thorpe et al. (1999)
**One Shank**	L_{Shank} **(mm)**	length of shank	245	Thorpe et al. (1999)
	m_{Shank} **(kg)**	mass of one shank	1.148	Isler et al. (2006)<br>Thorpe et al. (1999)
	D_{Shank}^{CoM} **(mm)**	distance between shank CoM and ankle joint	131.3	Isler et al. (2006)<br>Thorpe et al. (1999)
	L_{tibia} **(mm)**	length of tibia	245	Thorpe et al. (1999)
	L_{fibula} **(mm)**	length of fibula	227.7	Zihlman and Cramer (1978)<br>Thorpe et al. (1999)

Parameter		Description	Value	Source
**One Foot**	L_{Foot} **(mm)**	length of foot	214.4	Isler et al. (2006)<br>Thorpe et al. (1999)
	m_{Foot} **(kg)**	mass of one foot	0.654	Isler et al. (2006)<br>Thorpe et al. (1999)
	D_{Foot}^{CoM} **(mm)**	distance between foot CoM and heel	100.1	Isler et al. (2006)<br>Thorpe et al. (1999)
	H_f **(mm)**	height of foot	45.0	Wang and Crompton (2004)<br>Thorpe et al. (1999)
	L_{rf} **(mm)**	length of rear foot	30.5	Wang and Crompton (2004)<br>Thorpe et al. (1999)
	L_{mf} **(mm)**	length of middle foot	108.9	Wang and Crompton (2004)<br>Thorpe et al. (1999)
	L_{ff} **(mm)**	length of fore foot	75.0	Wang and Crompton (2004)<br>Thorpe et al. (1999)
	L_{fm} **(mm)**	length of the third metatarsal	66.5	Wang and Crompton (2004)<br>Thorpe et al. (1999)
L_{isc} **(mm)**		length of ischium	71.7	Kozma et al. (2018)<br>Thorpe et al. (1999)
β **(°)**		ischial angle	18	Kozma et al. (2018)<br>Thorpe et al. (1999)
